# Change in neutrophil to lymphocyte ratio during immunotherapy treatment is a non-linear predictor of patient outcomes in advanced cancers

**DOI:** 10.1007/s00432-019-02982-4

**Published:** 2019-07-31

**Authors:** Mingjia Li, Daniel Spakowicz, Jarred Burkart, Sandip Patel, Marium Husain, Kai He, Erin M. Bertino, Peter G. Shields, David P. Carbone, Claire F. Verschraegen, Carolyn J. Presley, Gregory A. Otterson, Kari Kendra, Dwight H. Owen

**Affiliations:** 1 Division of Hospital Medicine, Department of Internal Medicine, Ohio State University Wexner Medical Center, Columbus, OH USA; 2 Division of Medical Oncology, Department of Internal Medicine, Ohio State University Wexner Medical Center, 320 W 10th Ave, A450B Starling Loving Hall, Columbus, OH 43210 USA; 3 Department of Biomedical Informatics, Ohio State University Wexner Medical Center, Columbus, OH USA

**Keywords:** Immunotherapy, Immune checkpoint inhibitor, Prognosis, Neutrophil to lymphocyte ratio, NLR

## Abstract

**Background:**

The neutrophil to lymphocyte ratio (NLR) is known to be prognostic for patients with advanced cancers treated with immune checkpoint inhibitors (ICI), but has generally been evaluated as a single threshold value at baseline. We evaluated NLR at baseline and within first month during treatment in patients who received ICI for advanced cancer to evaluate the prognostic value of baseline and of changes from baseline to on-treatment NLR.

**Methods:**

A retrospective review of patients with advanced cancer treated with ICI from 2011 to 2017 at the Ohio State University was performed. NLR was calculated at the initiation of ICI and repeated at median of 21 days. Overall survival (OS) was calculated from the initiation of ICI to date of death or censored at last follow-up. Significance of Cox proportional hazards models were evaluated by log-rank test. Calculations were performed using the survival and survminer packages in *R*, and SPSS.

**Results:**

509 patients were identified and included in the analysis. Patients with baseline and on-treatment NLR < 5 had significantly longer OS (*P* < 0.001). The change in NLR overtime was a predictor of OS and was observed to be non-linear in nature. This property remained statistically significant with *P* < 0.05 after adjusting for age, body mass index, sex, cancer type, performance status, and days to repeat NLR measurement. Patients with a moderate decrease in NLR from baseline had the longest OS of 27.8 months (95% CI 21.8–33.8). Patients with significant NLR decrease had OS of 11.4 months (95% CI 6.1–16.7). Patients with a significant increase in NLR had the shortest OS of 5.0 months (95% CI 0.9–9.1).

**Conclusions:**

We confirmed the prognostic value of NLR in patients with advanced cancer treated with ICIs. We found that change in NLR over time is a non-linear predictor of patient outcomes. Patients who had moderate decrease in NLR during treatment with ICI were found to have the longest survival, whereas a significant decrease or increase in NLR was associated with shorter survival. To our knowledge, this is the first study to demonstrate a non-linear change in NLR over time that correlates with survival.

## Introduction

Immune checkpoint inhibitors (ICI) targeting cytotoxic T-lymphocyte-associated protein 4 (CTLA-4) and programmed cell dealth-1/program cell death ligand-1 (PD-1/PDL-1) proteins are able to augment host anti-tumor immune response (Kantarjian et al. 2016; Patel and Minn 2018). Despite the advent of new immunotherapies, there are few clinical prognostic tools available for patients who are offered treatment with ICIs. Previous studies have evaluated PD-L1 expression and tumor mutational burden (TMB) as potential biomarkers. These markers have shown great promise, but there are significant limitations due to cost, assay variability and tumor heterogeneity of PD-L1 expression (McLaughlin et al. 2016; Rimm et al. 2017), need for adequate tissue, requirement for invasive biopsy, and lack of standardization for interpretation of TMB (Rizvi et al. 2015; Carbone et al. 2017; Hellmann et al. 2018).

Cancer-associated inflammation leads to poor survival (Naqash et al. 2018; Hanahan and Weinberg 2011; Mantovani et al. 2008). Neutrophil to lymphocyte ratio (NLR) has been studied as a marker for systemic inflammation (Naqash et al. 2018). NLR has been demonstrated to be prognostic for cancer patients who have received ICI, with low baseline NLR at the start of ICIs being associated with favorable clinical outcomes (Naqash et al. 2018; Ameratunga et al. 2018; Lalani et al. 2018; Sacdalan et al. 2018; Lawati 2018; Park et al. 2018; Bagley et al. 2017; Zaragoza et al. 2015; Khoja et al. 2016). In past studies, the decrease in NLR during treatment was associated with improved survival. However, the decrease in NLR has not been quantitated. Instead, patients with decreasing NLR were combined into a single cohort with favorable OS. To evaluate whether there is any association between the degree of change and overall survival, we studied NLR at baseline and during ICI treatments in patients with advanced cancer at our institution.

## Patients and methods

We identified 509 patients with advanced cancer from 2011 to 2017 at the Ohio State University Comprehensive Cancer Center who received at least 1 dose of ICI as part of a retrospective study approved by the institutional review board at the Ohio State University.

### Data collection

Clinically relevant data were collected in REDCap after retrospective review of electronic medical records (Harris et al. 2009). Patients’ baseline complete blood count (CBC) with differential was collected on the day before receiving ICI or within 7 days prior to initiation of ICI. On-treatment CBC was collected at the next blood draw. If on-treatment CBC was less than 7 days from the initiation of ICI, a CBC closest to 14 days post-initiation of ICI was used. Clinical characteristics were extracted from the medical record including age at ICI, gender, cancer type and stage, performance status at time of ICI, BMI, and type of immunotherapy.

### Statistical analysis

NLR was defined as the absolute neutrophil count divided by the absolute lymphocyte count obtained from CBC. Overall survival (OS) was reported in number of months from the initiation of ICI to the date of death or last follow-up. All univariate and multivariate analyses were conducted using survminer package in R and IMB SPSS Version 25. Univariate analyses used Kaplan–Meier survival curves with log-rank test. Multivariate analyses used Cox proportional hazards models, defining the hazard function for each patient *k* as:

$$h_{\text{k}} \left( t \right) = h_{0} \left( t \right)e^{{\left\{ {\mathop \sum \limits_{t = 1}^{n} \beta_{1} N_{\text{B}} + \beta_{2} N_{\text{C}} + \beta_{3} N_{\text{C}}^{2} } \right\}}} ,$$

where *h*(*t*) is the hazard function at time *t* = 1 to *n*, *N*_B_ the baseline NLR value, *N*_C_ the change in NLR from baseline to treatment, and *N*_C_3 is the cubic change in NLR. The same method was used to control additional covariates, including BMI, age, sex, time to the second NLR measurement, Eastern Cooperative Oncology Group (ECOG) performance status, cancer, and immunotherapy treatment. Model evaluation, particularly to determine whether a polynomial change in NLR demonstrated significant improvement in the model and which exponentiation, was performed using a likelihood ratio test of nested models, implemented in the lmtest package in *R* (Kantarjian and Wolff 2016). Code to reproduce all analyses and figures can be found at https://github.com/spakowiczlab/nlr-io.

## Results

### Patient characteristics

Patient characteristics for the 509 patients are summarized in Table 1. There were 314 (61.7%) male and 195 (38.3%) female. The mean age was 62.7 years at the start of ICI, and 254 patients (49.9%) had melanoma, 111 (21.8%) had non-small cell lung cancer (NSCLC), 68 (13.4%) had renal cell carcinoma, 47 (9.2%) had head and neck cancer, 19 (3.7%) had bladder cancer, and 10 (2.0%) had sarcoma. The majority of patients had metastatic disease, with 69 (13.6%) patients treated for stage 3 cancer and 427 for stage 4. ICI treatment included nivolumab in 202 (39.7%) patients, ipilimumab in 163 (32.0%), pembrolizumab in 88 (6.7%), and nivolumab plus ipilimumab combination therapy in 34 (6.7%), other ICI 22 (4.3%).

**Table 1:** Patient characteristics

Total patients	509				
Male	314 (61.7%)	Baseline NLR	5.7 (mean)	Time to repeat lab	
Female	195 (38.3%)	Treatment NLR	6.24 (mean)	Median	21 days
Age	62.7 (mean)	BMI	28.86		
Cancer types		Stage		Immunotherapy	
NSCLC	111 (21.8%)	3	69 (13.6%)	Ipilimumab	163 (32.0%)
Melanoma	254 (49.9%)	4	427 (83.9%)	Nivolumab	202 (39.7%)
Renal cell Ca	68 (13.4%)	Other	13 (2.5%)	Pembrolizumab	88 (17.3%)
Head and neck	47 (9.2%)			Nivo + Ipi	34 (6.7%)
Bladder cancer	19 (3.7%)			Other	22 (4.2%)
Sarcoma	10 (2.0%)				

### Baseline NLR

**Fig. 1:**
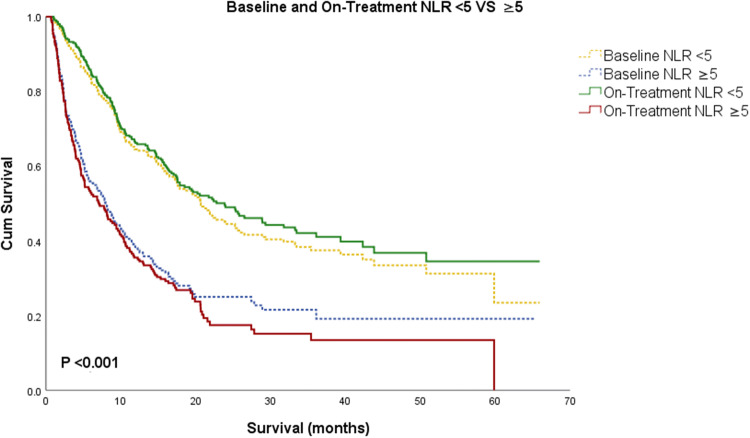
Kaplan–Meier survival analysis for patients with baseline is represented in dotted line and on-treatment in solid line. Patients were grouped based on NLR < 5 and NLR ≥ 5. Patients with lower NLR at baseline and on-treatment were associated with better prognosis

Patients with lower NLR at baseline were associated with improved OS. Patients were separated in two categories of NLR < 5 and ≥ 5, as previously done (Ameratunga et al. 2018; Park et al. 2018; Bagley et al. 2017; Capone et al. 2018; Sacdalan et al. 2018). Patients with baseline NLR < 5 had a median OS of 20.7 months (95% CI 17.1–24.5) compared to a median OS of 7.9 months (95% CI 5.8–10.1) in patients with baseline NLR ≥ 5 (*P* < 0.001, Fig. 1).

### On-treatment NLR

On-treatment NLR in our study was also associated with improved overall survival. The median time between baseline and on-treatment NLR was 21 days. Lower on-treatment NLR was associated with a significantly improved OS: 311 patients with on-treatment NLR < 5 had a median OS of 23.9 months (95% CI 17.6–30.1), whereas 198 patients with on-treatment NLR ≥ 5 had a median OS of 7.1 months (95% CI 4.7–9.4, *P* < 0.001, Fig. 1).

### Change in NLR during treatment

**Fig. 2:**
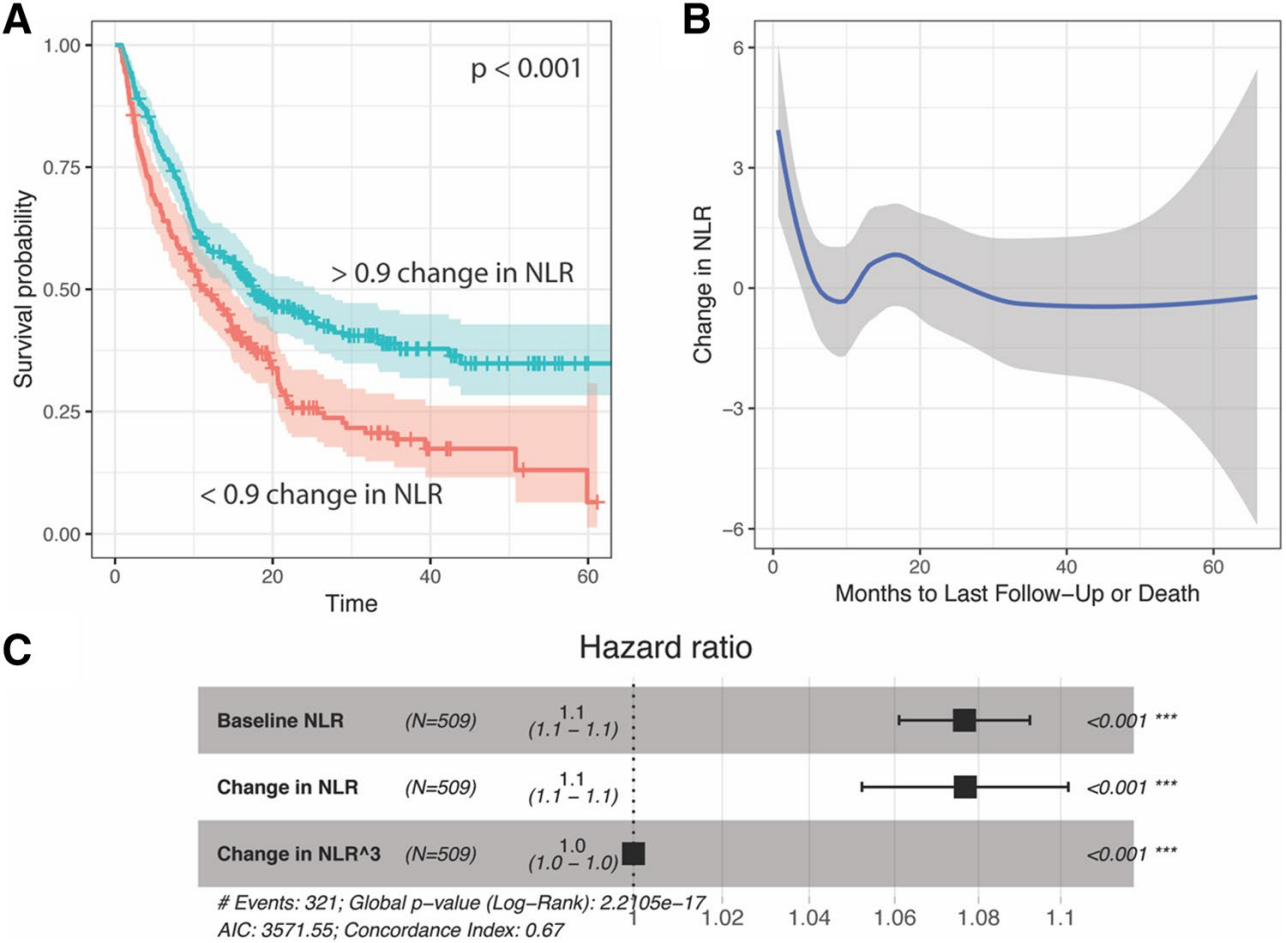
The change in NLR from baseline to on-immunotherapy measurements shows curvilinear character. **a** Survival curve stratifying by change in NLR of greater than 0.9. **b** Loess-smoothed fit of the change in NLR and months to last follow-up or death across several cancer types. **b** Cox proportional hazards model showing a significant polynomial term describing the change in NLR. The cubic term yielded a lower log likelihood than the quadratic change in NLR and the quadratic term did not significantly improve the model in addition to the cubic term

Following the observation that on-treatment NLR better stratified patient outcomes, we tested the hypothesis that the value of NLR was less important than its trend. Specifically, a starting NLR ≥ 5 was less important than whether it decreased on ICI treatment. The change in NLR was observed as a univariate Kaplan–Meier survival plot. A maximally selected rank test showed that the optimal stratum for the change in NLR was at 0.9, and indeed this value strongly stratified overall survival of patients (*P* < 0.001, Fig. 2a).

To rule out dependence on when the on-treatment NLR was measured (that is, the change in NLR could be gradual, and patients who were tested relatively early after the baseline NLR may show less change), we, therefore, visualized the change in NLR based on the time of the repeat test, and observed no relationship (slope not significantly different from 0, Fig. 2b). However, we noticed that the changes in NLR were not linearly related to survival. A loess fit showed strong curves at times of death < 12 months (Fig. 2c).

To evaluate whether the non-linear change in NLR significantly improves the accuracy of survival models, we pursued a multivariate Cox proportional hazards approach. In a model for overall survival that included the baseline NLR value, the change in NLR was also a significant predictor, as suggested by the survival plot (Fig. 2a). We explored this relationship further by observing the change in NLR with respect to the number of months to last follow-up or death (Fig. 2b). This showed strongly curvilinear features, where highly positive changes in NLR predict poor outcomes, and those with the longest survival showed a decrease in NLR of less than one. However, the addition of a polynomial on the change in NLR was also significant. A series of polynomials on the change in NLR were tested by likelihood ratio test of nested models, which demonstrated that a cubic term most improved the model (Fig. 2c). This cubic term remained a significant predictor when controlling for age, BMI, sex, ECOG performance status, cancer, and the type of immunotherapy received. NLR is, therefore, the best fit to outcome non-linearly, in the sense that the relationship is not a straight line, but rather a curvilinear relationship or polynomial linear function.

**Fig. 3:**
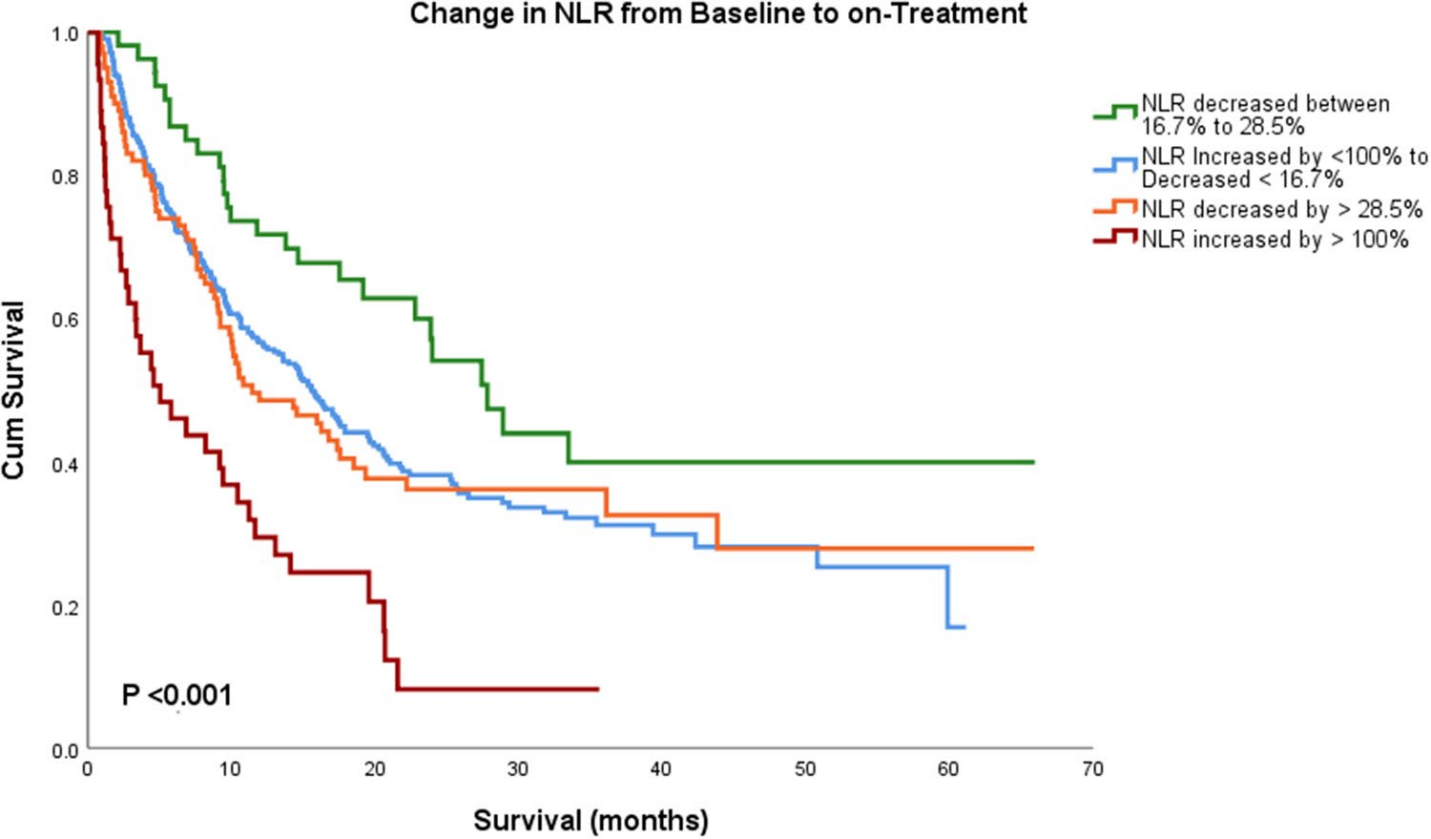
Kaplan–Meier survival analysis showing change in NLR from baseline to on-treatment based on the percentage of change. Patient with modest decrease in NLR had the longest median OS

This effect was apparent when we stratified the cohort by their change in NLR. Using semi-arbitrary strata, 45 patients showed a large increase in NLR by more than 100%, 311 patients changed little (as defined by increase less than 100% to decrease less than 16.7%), 53 patients decreased moderately (16.7%–28.5%), and 100 patients showed a large decrease (beyond 28.5). Patients with a moderate decrease in NLR (between 16.7% and 28.5%) had the longest OS with median OS of 27.8 months (95 CI 21.8–33.8), whereas patients with decrease in NLR greater than 28.5% had OS of 11.4 months (95 CI 6.1–16.7) months. Those who had significant increase in NLR by more than 100% during treatment had the shortest OS at 5.03 (95 CI 0.94–9.1) months (overall *P* < 0.001, Fig. 3).

## Discussion

In the era of cancer immunotherapy, indications for ICI use in cancer care are expanding at a rapid pace (Antonia et al. 2017; Marin-Acevedo et al. 2018). Previous studies explored high PDL-1 expression and tumor mutation burden (TMB) as potential predictive biomarkers. Patients with high tumor expression of PD-L1 tend to have a better outcome (Garon et al. 2015; Gettinger et al. 2014; Reck et al. 2016). However, the durable benefit seen in some patients without PD-L1 expression limits its role as an exclusionary prognostic biomarker. TMB has also shown promise (Rizvi et al. 2015; Carbone et al. 2017; Hellmann et al. 2018). However, there is a lack of standardization for TMB calculation, a variety of assays available, and not all tumor mutations will lead to altered proteins that are measured by TMB (Rizvi et al. 2015; Carbone et al. 2017). Furthermore, both PDL-1 expression and TMB analysis require expensive and invasive biopsies to obtain tissue samples, which are impractical to use for frequent biomarker monitoring. Recent evidence has suggested the microbiome as an additional marker, though it has not been widely adopted (Routy et al. 2018).

In this study, we confirmed the prognostic value of NLR at the initiation of ICI treatment. Interestingly, the relationship between NLR and survival was non-linear. Only a moderate decrease in NLR was associated with the longest OS. An excessive decline in NLR was associated with reduced OS.

NLR is calculated from existing routine labs for patients who are receiving ICI and it is obtained with a routine peripheral blood draw. The inexpensive and readily available nature of NLR allows it to be conveniently monitored overtime. The change in NLR is a useful biomarker to the extent that it can be calculated in a timely manner in the clinic. Ideally, the baseline NLR value would stratify patients who will respond to ICI vs. those who are better suited to alternative treatments such as chemotherapy. More study is needed to validate this prediction, and if it holds, to identify the optimal time for repeat NLR.

The mechanism by which NLR relates to ICI activity and OS is unknown. Our results show that large increases or decreases are negatively associated with OS. Large increases in NLR may reflect increased tumor burden, lack of ICI efficacy and, therefore, decreased OS. Large decreases in NLR are more challenging to interpret. We observed that change in NLR is driven primarily by the decline in neutrophils and that the lymphocyte count remained relatively constant in our patient population. The common causes of decline in neutrophils can include decreased bone marrow activity, infection, and malnutrition, all of which have been shown to impact OS (Bouteloup et al. 2017). Agranulocytosis and neutropenia may also rarely occur as a result of auto-immune toxicity from ICI treatment (Barbacki et al. 2018).

This is the first study to demonstrate the non-linear relationship between change in NLR and OS during ICI treatment. There are several limitations in our study, including its retrospective nature, unknown mechanism of action, inclusion of a heterogeneous patient population, and the inability to evaluate variables with known predictive power in this context, notably the line of therapy and mutation status. Further studies looking into relationship between NLR and tumor micro-environment, medication interactions, infection, nutrition status, microbiota, line of therapy, and immune-related adverse events may help to delineate the mechanisms between the non-linear change in NLR and OS. Prospective studies with a larger patient cohort are needed for validation.

## Conclusion

This is the first study to demonstrate the non-linear relationship between change in NLR and survival during ICI treatment for patients with advanced cancer. Further studies looking into the reasons for this non-linear relationship, including possibly the contributions of tumor micro-environment, infection, nutrition status, microbiota, and other immune-related adverse events to change in NLR may help to delineate the mechanisms between the non-linear change in NLR and clinical outcomes.

## Data Availability

The datasets used and/or analyzed during the current study are available from the corresponding author on reasonable request.

## Abbreviations

CBC: Complete blood count
CTLA-4: Cytotoxic T-lymphocyte-associated protein 4
ECOG: Eastern cooperative oncology group
ICI: Immune checkpoint blocker
NLR: Lymphocyte to neutrophil ratio
OS: Overall survival
PD-1: Programmed cell dealth-1
PDL-1: Program cell death ligand-1
TMB: Tumor mutation burden
